# A Picture is Worth How Many Words? The Impact of Visual Cues on the Story Retelling of Children with and without ADHD

**DOI:** 10.1007/s10802-026-01494-3

**Published:** 2026-08-20

**Authors:** Hend Samniya, Ricky Haneda, Gail Tripp

**Affiliations:** 1 Okinawa Institute of Science and Technology (OIST) Graduate University, Okinawa, Japan; 2 University of California, Riverside, USA

**Keywords:** ADHD, Narrative, Story retelling, Memory load, Language skills, Discourse analysis

## Abstract

Children with attention deficit hyperactivity disorder (ADHD) evidence poorer language skills than their neurotypical (NT) peers, including their narrative (storytelling) abilities. Available narrative studies include a circumscribed number of variables, with limited attention to the effect of task demands on performance. We compared the story retelling skills of children with and without ADHD under different memory loads. Ninety-six 8-12-year-old children, 50 meeting DSM-5-TR criteria for ADHD (54.9% boys) and 46 NT children (39.1% boys) completed a story retelling task under two conditions, i.e., with and without visual story cues. The children’s stories were coded across a range narrative dimensions, with performance compared across groups and conditions. Children with ADHD produced less complete and coherent stories that included higher rates of dysfluencies and linguistic errors than NT children. Across groups, performance was better in the presence of visual story cues, with larger effects for the ADHD group on some variables. Findings highlight the extent of narrative production difficulties in children with ADHD and the positive effects of visual cues. Given the importance of narrative skills for communication and social interactions, their inclusion in the comprehensive assessment of children with ADHD is recommended.

**Supplementary Information:**

The online version contains supplementary material available at https://doi.org/10.1007/s10802-026-01494-3.

## Introduction

In addition to impairing levels of inattention and/or hyperactivity/impulsivity, children with attention deficit hyperactivity disorder (ADHD) evidence poorer language skills than their neurotypical (NT) peers. Several studies report children with ADHD obtain lower scores on standardized measures of receptive, expressive and pragmatic language (for reviews see Green et al., 2014; Korrel et al., 2017). Less is known about the discourse skills of children with ADHD, with most studies focusing on their storytelling (narrative) abilities (see Carruthers et al., 2022; Hill et al., 2026; Jepsen et al., 2022 for reviews). Narrative skills play a critical role in social communication, allowing the speaker to connect with others through reporting on events and sharing personal experiences (Mandelbaum, 2012; Schegloff, 1997). Delivering a complete story relies on the coordination of several elements of narration^1^, including topic maintenance, event sequencing, explicitness, coherence, referencing, cohesion, and fluency (Bliss et al., 1998). Storytelling also requires grammatical (morphosyntax) accuracy and sufficient vocabulary to convey meaning clearly (Lockhart, 2004; Nuroh & Syahid, 2023).

Several studies report children with ADHD experience difficulty with narrative completion (explicitness) compared to their NT peers during both story generation (Derefinko et al., 2009; Flory et al., 2006; Freer et al., 2011; Leonard et al., 2009; Luo & Timler, 2008; Renz et al., 2003; Rumpf et al., 2012) and retelling tasks (Bergman & Hallin, 2021; Lorch et al., 1999, 2010; Papaeliou et al., 2015; Purvis & Tannock, 1997; Tannock et al., 1993). Difficulties with topic maintenance (Flory et al., 2006; Fortea et al., 2018; Zenaro et al., 2019), sequencing (Flory et al., 2006; Lorch et al., 2010; Purvis & Tannock, 1997; Tannock et al., 1993), coherence (Freer et al., 2011; Jepsen et al., 2024, 2025; Lorch et al., 2010), referencing (Jepsen et al., 2025; Kuijper et al., 2015; Lorch et al., 1999; Tannock et al., 1993), fluency (Kuijper et al., 2017; Lee et al., 2017) and semantics (Purvis & Tannock, 1997; Tannock et al., 1993) are also reported in the narratives of children with ADHD. Findings on morphosyntax (errors and/or complexity) and narrative cohesion are mixed (Bergman & Hallin, 2021; Fortea et al., 2018; Jepsen et al., 2025; Kuijper et al., 2017; Rumpf et al., 2012; Tannock et al., 1993). The number of studies addressing some elements of narration is small, and the number of elements tested within individual studies limited. Together this hampers the establishment of a comprehensive (broad) profile of the narrative skills of children with ADHD compared with their TD peers (Jepsen et al., 2025).

The mechanisms underlying the poorer performance of children with ADHD in narrative tasks are not well understood. It is not clear if observed differences are the result of the children’s ADHD symptoms (e.g., deficits in sustained attention), their generally poorer language skills, comorbidity with learning or other disorders, or other aspects of their cognitive functioning (e.g., the load the task imposes on working memory (WM). In general, narrative studies have not excluded, or controlled for, comorbid conditions or executive function performance. Working memory is thought to play an important role in language processing by temporarily storing and manipulating linguistic information needed for tasks including understanding sentences, maintaining coherent conversations, and learning new vocabulary (Baddeley, 1992, 2003). Additionally, the control system that manages and coordinates the elements of working memory is purported to be responsible for the integration of linguistic information with context, facilitating more complex language functions like inference-making and discourse comprehension (Baddeley, 2003; Levelt, 1981, 1983). Interestingly, Jepsen et al. (2025) recently reported executive function, i.e., working memory updating and inhibition, did not mediate the association between ADHD and narrative language production in 7–11 old children.

Despite the assumed contribution of working memory to narrative skills (Veraksa et al., 2020), and evidence of poorer working memory in children with ADHD (Christoforou et al., 2023), few studies have assessed the impact of memory load on narrative performance. There is some indirect evidence that the provision of visual supports may reduce group differences in story retelling. Luo and Timler (2008) reported no differences in the narrative organization of children with and without ADHD when story pictures were provided. In the absence of such supports, Lorch et al. (2010) and Papaeliou et al. (2015) reported children with ADHD recalled less information and were less sensitive to story meaning, than NT children.

We identified a single study that directly compared story retelling with and without visual supports (pictures). Bergman and Hallin (2021) reported visual supports differentially affected story retelling in adolescents with ADHD and their NT peers. In the no visual support condition, the stories of the ADHD group were significantly shorter with higher rates of grammar errors. Performance on these variables was similar across groups in the presence of story pictures. Children in the NT group produced longer, more complex, sentences in the picture condition compared with the no picture condition. This effect was not seen in the ADHD group. Both groups included more key story elements when telling a story without pictures.

These findings suggest providing story pictures reduces the cognitive demand or load imposed by the retelling task (Sweller, 2010)^2^. In the presence of visual supports, participants are not required to recall the story content or order (intrinsic load), presumably freeing up resources for deeper story comprehension and a greater focus on story production (germane load). Bergman and Hallin’s (2021) finding that both groups included more story elements in the absence of story pictures raises the possibility that these visual supports might also increase the extraneous task load. Given the reported lower working memory skills of those with ADHD, visual cues might be expected to improve their task performance more than that of NT children.

The current study extends research on the effect of story pictures on the narrative skills of children with ADHD and their NT peers. Specifically, the narrative structure, fluency, and linguistic accuracy of the children’s retold stories, are compared in the presence and absence of visual story cues. Based on prior research, children with ADHD are expected to perform less well on the story retelling task than their NT peers (i.e., poorer narrative completion, coherence and fluency, and more errors). The effect of visual cues on narrative performance is more difficult to predict. Theory, and the available evidence, suggest the narratives of children with ADHD will improve in the presence of visual cues, possibly reaching the level of their NT peers, but see Jepsen et al. (2025). The findings of Bergman and Hallin (2021) suggest the performance of NT children will also benefit from visual cues, perhaps not to the same extent as in children with ADHD, as they will likely already perform better in the no-cue condition.

A detailed quantitative coding scheme was developed for the study to code the children’s story retelling performance, covering a wide range of narrative dimensions (topic maintenance, event sequencing, explicitness, coherence, referencing, cohesion, fluency, semantics, morphosyntax, and phonology). The breadth of the coding scheme designed to provide a more comprehensive profile of the children’s narrative skills than previous studies.

## Method

Ethical approval for the study was obtained from the Okinawa Institute of Science and Technology Graduate University (OIST) Human Subjects Research Review Committee (reference number HSR-2021-008). All participants were volunteers. Parents and teachers provided written consent and the children written assent to participate.

### Participants

Participants were 8-12-year-old children meeting DSM-5-TR diagnostic criteria for ADHD^3^ (*n* = 50, 56% boys) and a group of NT children (*n* = 46, 39% boys). The majority of children were described as white (ADHD group 64%; NT group 52.2%) or mixed race (ADHD 26%; NT 41%) by their parents. Over half the participating families reported a household income of more than US$60,000 (ADHD 61%; NT 65%). Six children in the ADHD group met DSM-5 criteria for oppositional defiant disorder (ODD), five for an anxiety disorder and one for depression. Within the NT group three children meet criteria for an anxiety disorder. The groups were not significantly different on any of these demographic variables (see Table 1). Within the ADHD group, 28 children met criteria for inattentive presentation, five hyperactive/impulsive presentation, and 18 combined presentation ADHD. Children prescribed stimulant medication (*n* = 17) discontinued its use for 48 h prior to participation, non-stimulant medication (*n* = 1) was not withheld.

**Table 1:** Demographic characteristics of the ADHD and neurotypical (NT) groups

Variable	ADHD				NT					
	*n*	*M*	*SD*	Range	*n*	*M*	*SD*	Range	t/χ2	*p* value
Age (months)	50	121.14	18.74	95-155	46	119.83	20.18	96-155	0.33	0.741
Symptom severity^a^	50				45					
Inattention		16.82	5.19	8-26		6.71	3.99	0-15	10.71	<0.001
Hyperactivity/		13.08	5.31	3-26		5.59	3.79	0-14	7.96	<0.001
Impulsivity										
Sex (F/M)			23/27				28/18		2.13	0.145
SES										
Primary caregiver education level^b^ *n*	50		3/3/13/23/8		41		4/0/6/16/15		7.97	0.093
1/2/3/4/5										
Total household income^c^ *n*	49		3/16/30		40		0/14/26		3.35	0.187
1/2/3										
Ethnicity										
Black/White/Asian/multiracial *n*	50		1/32/4/13		46		0/24/3/19		2.79	0.425
Comorbidity *n*										
ODD			6				0			
Anxiety			5				3			
Depression			1				0			
Medicated *n*										
Yes			17				-			
No			32				-			
In the past			1				-			

Missing data: symptom severity (NT, *n* = 1), caregiver education (NT, *n* = 5), household income (ADHD, *n* = 1; NT, *n* = 6); ODD = oppostional defiant disorder

^a^  = Symptom severity scores = sum of DSM-5-TR item scores from Conners Behavioral Rating Scale (CBRS) in the ADHD group, and the Conners-3 in the NT group

^b^  = Parent education level: 1 = high school/GED. 2 = technical school. 3 = some college. 4 = bachelor’s degree. 5 = graduate school

^c^ = Household income: 1 = less than US$30,000. 2 = US$30,000 - US$60,000. 3 = more than US$60,000

Inclusion criteria for all children were: English as a first language, aged 8 to 12 years, Full Scale (FSIQ, ADHD group) or estimated Short Form (SFIQ^4^, NT group) IQ ≥ 80, normal or corrected vision, no current or history of head injury, neurological disorder, psychosis, or diagnosis of a language disorder^5^, speech sound disorder, stuttering, or autism spectrum disorder. Children in the ADHD group were required to meet the DSM-5TR diagnostic criteria for ADHD; those in the NT group to demonstrate fewer than four parent-reported symptoms of inattention or hyperactivity/impulsivity.

All children were recruited through the OIST Children’s Research Center. Families of children in the ADHD group received study information from American (US) school personal, health care professionals, community support organizations, and the Center website. Neurotypical children were recruited through outreach to parents of children attending US and international schools, through public events, and the Center website.

### Procedure

#### Diagnostic, Cognitive and Language Assessments

All children in the ADHD group completed multi-method, multi-informant research diagnostic assessments for the current study. Their parents and teachers completed the Conners Comprehensive Behavioral Rating Scale forms^6^ (Conners 2008a) and questions about the child’s academic and social functioning.

Parents also completed developmental and medical history questionnaires, a clinical interview about their child’s daily functioning, and the ADHD^7^, oppositional defiant disorder (ODD) and conduct disorder (CD) sections of the K-SADS-PL for DSM-5 (Kaufman et al., 2016). The children were interviewed about their school progress, friendships and behavior, and completed cognitive assessments, including the Wechsler Intelligence Scale for Children, 5th edition (10 primary subtests, WISC-V, Wechsler, 2014). Interviews and cognitive assessments were carried out by a US (PhD) licensed clinical psychologist and supervised research staff (experienced Masters’ level US licensed school and counselling psychologists) over two consecutive mornings. Data from all sources, including child observations during the cognitive and language assessments^8^, was used to determine if children met the DSM-5-TR criteria for ADHD; symptoms were not summed across informants. Diagnostic decisions were reviewed by two doctoral-level US licensed clinical psychologists.

Children in the NT group completed abbreviated diagnostic and cognitive assessments. One parent in each family completed the Conners-3 Rating Scale^5^ (Conners 2008b) and abbreviated demographic and health questionnaires. The children completed the Digit Span, Vocabulary, and Matrix Reasoning subtests from the WISC-V. The Digit Span subtest scaled score was used as a measure of working memory (across groups). Estimated SFIQ scores^9^ were calculated from the sum of the scaled scores for the Vocabulary and Matrix Reasoning subtests, using the procedure described by Tellegen and Briggs (1967) This approach is recommended as it takes account of the reliabilities of the included subtests (Sattler, 2024). The reliability *r*_*xx*_ = 0.91 and validity *r* = .83 of the Vocabulary and Matrix Reasoning short form are good compared with the WISC-V FSIQ (Sattler, 2024). Participant demographic and diagnostic characteristics, together with between group comparisons, are presented in Table 1.

To screen for general language skills, all children were administered the age-appropriate Core Language subtests, making up the Core Language Score (CLS) of the 5th edition of the Comprehensive Assessment of Language Fundamentals (CELF-5, Wiig et al., 2013)^10^. This measure was administered and scored by a member of the research team, a qualified speech language pathologist. Table 2 presents the cognitive and language measure scores for the ADHD and NT groups, together with between-group comparisons.

**Table 2:** Descriptive statistics and group comparisons for the cognitive and language assessment measures

Variable	ADHD				NT					
	*n*	*M*	*SD*	Range	*n*	*M*	*SD*	Range	*t*	*p* value
Cognitive										
Full Scale IQ	50	102.16	11.35	80-135	-	-	-	-		
Estimated IQ	50	103.70	8.96	88-126	45	108.44	13.11	82-129	-2.04	0.045
WM^a^	50	9.00	2.70	4-16	45	11.42	2.86	5-17	-4.25	<0.001
Language										
CELF-5^b^	50	99.86	12.21	80-127	45	108.51	11.09	90-133	-3.6	<0.001
Missing data: Estimated IQ (NT, *n* = 1), WM (NT, *n* = 1), CELF-5 (NT, *n* = 1)										

^a^ = Working memory score = scaled score from the Digit Span subtest from the WISC-V

^b^ = Age-appropriate Core Language Score (CLS)

#### Experimental Task

All participating children completed a narrative task^11^ in which they were asked to listen to, and then retell, two short stories, one with and one without visual story cues. These stories were “The Greedy Dog” and “The Bald Man and the Fly”, Aesop’s fables of similar length and complexity, containing the same number of plot units (see Table S2 in Supplemental Materials). The stories were presented on a computer monitor with sequenced illustrations (6 per story), narrated by a male speaker with a soft Midwest American accent (see Fig.S1 and Fig.S2 in Supplemental Materials for the story pictures). The same examiner administered the task to all participating children. The task protocol and participant instructions are available in the Supplemental Materials. Generally, no feedback from the examiner was allowed during story retelling.

The children were presented with one story at a time and asked to retell the story immediately after hearing it. In one condition, they retold the story while the illustrations were re-presented (cue condition). In the other condition without the illustrations (no-cue condition). To control for possible practice and story effects, condition and story order were counterbalanced, i.e., within each group, half of the children listened to the “Greedy Dog” story first, and amongst those, half completed the visual cue condition first. The children’s narratives were recorded and transcribed for coding (transcription instructions are available in the Supplemental Materials). Transcripts, rather than audio recordings, were coded as these provide a standardized textual record, ensuring all coders review the same linguistic data under identical conditions, a prerequisite for dependable reliability coefficients.

### Coding Scheme

The transcripts were coded using a scheme developed for the current study, informed by the language dimensions referenced in the Narrative Assessment Profile (NAP; Bliss et al., 1998). This included the narrative elements of topic maintenance, event sequencing^12^, explicitness and coherence, referencing, cohesion, and fluency. In the NAP, these elements are treated qualitatively. For the current study, we added quantitative measures for these variables. Phonology, semantic, and morphosyntax error variables, were also included. All included variables were categorized under the dimensions of “narrative structure”, “fluency”, or “linguistic accuracy” (see Table 3 for details). The number of occurrences of each variable was recorded, except for global coherence which was rated on a 5-point scale. A composite “informativeness” variable was created by combining the number of correctly included events, basic facts, plot units and characters/objects.

The coding scheme relies on observable, descriptive output and classifies errors based on the objective relationship between the target and the uttered word (Dell, 1986; Levelt, 1993). Any deviation from the standard form was coded as an error, irrespective of whether it represented a final produced error, or a self-correction. The coding scheme is included in the Supplemental Materials.

**Table 3:** Narrative variables, categories and descriptions

Category	Variable	Description	Example of an error
Narrative structure	Off-topic deviations	Diversions from the main subject or storyline and addition of false events.	“The dog was crossing a bridge with a piece of meat in his mouth. *He stopped to bark at a cat on the bridge*. Then, he let go of his own piece of meat and attacked the other dog…”
	Achronological presentation of events^a^	Events presented out of their chronological order.	“…The dog lost his own piece of meat because the stream swept it away. Then he jumped in the water to attack the other dog…”
	Events presented^b^	Inclusion of key incidents in the story.	“The dog was crossing a bridge over a stream with a piece of meat in his mouth. He saw another dog in the water with a piece of meat bigger than his. He wanted to get the other piece of meat but, the stream swept his meat away”. Here, the event of letting go of the meat and jumping into the water is omitted.
	Basic facts presented^b^	The delivery of essential details and information within the story.	“The dog was crossing a bridge over a stream with a piece of meat in his mouth. He saw another dog in the river. He let go of his piece of meat and he jumped into the river to get the bigger piece of meat. Unfortunately, the stream swept his meat away” Here, the information about the other dog holding a piece of meat is omitted.
	Story units presented^b^	Inclusion of story units that make up the narrative structure. In this study the units are ‘introduction’, ‘problem’, ‘attempt at solution’, and ‘realization’.	“A dog was crossing a bridge with a piece of meat in his mouth, he saw another dog in the water with a piece of meat twice as big as his, he let go of his own meat and jumped into the water to attack the other dog and get his piece of meat. His piece of meat was swept by the river” Here, the realization unit is missing.
	Characters/objects introduced^b^	Initial mention of people and essential items.	“He was crossing the bridge with a piece of meat in his mouth…”
	Elaborations to engage the listener	Additional details added to capture the listener’s interest.	“…He jumped into the water to get the other piece of meat, what a greedy dog…”
	Global coherence rating	A measure of how logically and cohesively the story elements are connected.	
Fluency	False starts	Rephrasing or restarting the introduction of the story.	“ A dog was crossing a stream, I mean a dog was crossing a bridge…”
	Self-interruptions	When the speaker halts their speech to make corrections or changes.	“…He let of his m- his piece of meat…”
	Pauses	Moments of silence exceeding 2–3 s within speech.	“ The dog was (4 s pause) crossing the bridge…”
	Repetitions	Repeating words or fragments of words.	“…The dog realized, the dog realized that he lost his piece of meat…”
	Fillers	Use of non-meaningful words or sounds (e.g., um, uh, like).	“…He um jumped into the river to get the other piece of meat…”
Linguistic accuracy	Referencing errors	Mistakes in pronouns and referring to a character or object with the wrong name, or vague referencing.	“*She* was crossing a bridge with a piece of meat in *his* mouth…”
	Cohesion errors	Mistakes or omission in using linguistic elements that link sentences or phrases together.	“…He saw his own shadow in the water, *because* he thought it was another dog…”
	Phonological errors	Mistakes in the sound system of language.	“ A *dod* was crossing a bridge…”
	Semantic errors^c^	Inaccurate use of words like referring to characters, objects, actions, or adjectives.	“ A dog was crossing a bridge with a piece of *bone* in his mouth”
	Morphosyntax errors^c^	Mistakes in the grammatical structure of words and sentences.	“…with a piece *more bigger* than his”

^a^ = The achronological events variable was not included in the statistical analyses due to the low number of occurrences

^b^ = These variables were combined to create the variable ‘informativeness’

^c^ = These variables were combined in the analysis due to severe skewness and kurtosis of the separate error scores

### Coder Training and Reliability

Two trained raters, blind to participant group membership, age, and assigned sex coded the transcripts. One rater coded all 96 transcripts, the second rater 24 (25.0%) transcripts, selected at random, to assess inter-rater reliability. Coders completed four training sessions: (1) review of coding guidelines, (2) practice coding, (3) independent coding of two transcripts followed by consensus discussions, and (4) independent coding of two additional transcripts to verify agreement. Training transcripts were supplemental to the study transcripts. Coder reliability was calculated with Krippendorff’s alpha for most narrative variables. This reliability measure is recommended for communication research and text analysis (Hayes & Krippendorff, 2007; Krippendorff, 2004). Reliability was satisfactory with coefficients ranging from 0.79 (achronological events) to 1.00 (false starts). Intraclass correlation coefficients (ICC)^13^ were calculated to assess the global coherence ratings (Koo & Li, 2016). The ICC for coherence was 0.97 (see Table S3 in Supplemental Materials for all reliability coefficients).

### Data Preparation and Analytic Plan

Preliminary analyses indicated the length of the ADHD group narratives were shorter than those of the NT group. To control for narrative length, variables measured at the word level were converted to rates, i.e., target word per total words (Balčiūnienė & Kornev, 2024; Samniya et al., 2025). The distributions of all narrative variables were checked for skewness, kurtosis, and the presence of extreme outliers. As necessary, log transformations were used to correct the distributions (self-interruptions, pauses, repetitions, fillers, referencing errors, cohesion errors, semantic and morphosyntactic errors) (Zar, 2014). Two-way mixed ANOVA (group by condition) were used to analyze data meeting criteria for parametric analyses. Where data transformation did not improve distributions sufficiently, non-parametric analyses (Mann-Whitney U, Wilcoxon Signed Ranks, Chi Square, and McNemar’s test) were undertaken (off-topic deviations, false starts, phonological errors).

Independent t-tests indicated Estimated SFIQ, CELF-5^14^, and working memory scores were significantly lower in the ADHD group than the NT group (Table 2). These scores were correlated with the narrative variables. Correlations between Estimated SFIQ and the narrative variables were typically small and nonsignificant, suggesting Estimated SFIQ did not covary with performance on the narrative tasks (see Table S4 in Supplemental Materials) and was not controlled for in the analyses. Correlations between CELF-5 scores and the narrative variables were small to moderate (see Table S5 in Supplemental Materials), this is not unexpected given the shared measurement of underlying linguistic constructs (e.g., grammar, semantics, coherence), hence CELF-5 language scores were not treated as a covariate in the analyses. For working memory, correlations with the narrative variables were also small to moderate, sometimes with different patterns across groups (Table S6 in Supplemental Materials). As the study investigated the effects of visual cues/memory load on narrative performance, working memory was not treated as a covariate in the analyses.

To control for multiple comparisons Holm’s correction (McLaughlin & Sainani, 2014) was applied to families of narrative variables (parametric analyses only), combining *p* values across main effects and interactions. Results are presented with the original *p* values, the effects of correcting for multiple comparisons reported.

Where significant group effects survived correction for multiple comparisons, exploratory hierarchical multiple regression analyses were carried out to assess the independent contributions of working memory (WISC-V digit span), and structural language skills (CELF-5 CLS) to narrative performance, over and above ADHD symptom severity, separately for the two conditions.

## Results

### Cognitive and Language Skills

Children in the ADHD group obtained lower estimated SFIQ (*t*(93) = -2.04, *p* = .045, *d* = − 0.43) and working memory scores (*t*(93) = -4.25, *p* < .001, *d* = − 0.87) than their NT peers. Their performance on the CELF-5 CLS was also significantly lower than that of the NT group, (*t*(93) = -3.60, *p* < .001, *d* = − 0.74). For both measures the scores of the children in the ADHD group were within the normal range.

### Narrative Task Performance

Descriptive statistics and group comparisons for the narrative variables are presented in Table 4 (narrative structure variables), and Table 5 (fluency and linguistic accuracy word-level variables).

**Table 4:** Descriptive statistics for the narrative structure variables (Mean, SD, Median and Range)

			ADHD (*n* = 50)			NT (*n* = 46)			
Variable	Condition		*M*(*SD*)	Mdn	Range	*M*(*SD*)	Mdn	Range	Significance
Speech quantity									
Word count	VC		**79.96(18.85)**	81.00	31.00-121.00	**83.07(17.55)**	82.00	56.00-134.00	ADHD < NT
	noVC		**72.48(19.95)**	73.00	19.00-116.00	**82.15(14.89)**	82.00	49.00-122.00	
Narrative structure									
Off-topic deviations	VC		0.38(0.64)	**0.00**	0.00-2.00	0.24(0.52)	**0.00**	0.00-2.00	NS
	noVC		0.54(0.84)	**0.00**	0.00-3.00	0.33(0.63)	**0.00**	0.00-3.00	
Informativeness	VC		**14.96(1.85)**	15.50	9.00-17.00	**15.89(1.37)**	16.00	12.00-17.00	ADHD < NT VC > noVC Significant Interaction
	noVC		**13.54(2.89)**	15.00	4.00-17.00	**15.67(1.85)**	16.00	9.00-17.00	
Elaborations to engage listener	VC		**2.14(1.16)**	2.00	0.00-5.00	**2.30(1.09)**	2.00	0.00-5.00	NS
	noVC		**2.14(1.29)**	2.00	0.00-5.00	**2.20(1.02)**	2.00	0.00-4.00	
Coherence ratings (1-5)	VC		**3.58(1.11)**	4.00	1.00-5.00	**4.09(0.72)**	4.00	2.00-5.00	ADHD < NT VC > noVC Significant Interaction
	noVC		**2.76(1.04)**	3.00	1.00-5.00	**3.70(0.96)**	4.00	1.00-5.00	

VC = visual cue condition, noVC = no visual cue condition. Bolded means indicate parametric analyses (ANOVA) were undertaken, bolded medians indicate the nonparametric tests Mann-Whitney and Wilcoxon Signed Rank tests were used. Details of statistical comparisons are presented in the text

**Table 5:** Descriptive statistics for the fluency and linguistic accuracy variables rates (Mean, SD, Median and Range)

Variable (untransformed rates)		ADHD (*n* = 50)			NT (*n* = 46)			
	Condition	*M*(*SD*)	Mdn	Range	*M*(*SD*)	Mdn	Range	Significance
Fluency								
Self-interruptions	VC	**4.76(4.12)**	4.08	0.00-17.95	**3.74(3.47)**	2.95	0.00-16.87	ADHD > NT
	noVC	**5.40(3.49)**	5.13	0.00-14.29	**3.61(2.73)**	2.94	0.00-11.11	
Pauses	VC	**2.68(3.00)**	1.73	0.00-12.90	**0.79(1.58)**	0.00	0.00-6.45	ADHD > NT
	noVC	**3.29(3.21)**	2.59	0.00-12.20	**1.09(1.49)**	0.00	0.00-6.32	VC < noVC
Repetitions	VC	**3.13(2.91)**	2.31	0.00-14.53	**2.65(2.74)**	1.77	0.00-13.25	ADHD > NT
	noVC	**3.87(2.87)**	3.32	0.00-12.50	**2.62(2.32)**	2.19	0.00-10.91	
Fillers	VC	**1.16(1.60)**	0.00	0.00-6.15	**1.53(2.02)**	1.07	0.00-10.45	NS
	noVC	**1.56(2.33)**	0.00	0.00-11.63	**1.96(1.91)**	1.29	0.00-8.70	
Linguistic accuracy								
Referencing errors	VC	**1.06(1.71)**	0.00	0.00-9.68	**0.71(0.94)**	0.00	0.00-3.17	ADHD > NT
	noVC	**1.78(2.73)**	1.20	0.00-15.79	**0.63(0.92)**	0.00	0.00-3.26	
Cohesion errors	VC	**1.26(1.38)**	1.02	0.00-4.62	**0.37(0.65)**	0.00	0.00-2.11	ADHD > NT
	noVC	**1.88(1.88)**	1.34	0.00-8.33	**0.53(1.04)**	0.00	0.00-5.41	VC < noVC
Grammar + semantic errors	VC	**1.92(1.75)**	1.52	0.00-7.25	**0.65(1.03)**	0.00	0.00-5.45	ADHD > NT VC < noVC
	noVC	**3.36(3.73)**	2.60	0.00-21.05	**0.68(1.04)**	0.00	0.00-3.00	
Phonological errors	VC	0.37(0.73)	**0.00**	0.00-3.16	0.07(0.37)	**0.00**	0.00-2.41	ADHD > NT in VC
	noVC	0.23(0.82)	**0.00**	0.00-5.26	0.08(0.30)	**0.00**	0.00-1.28	

VC = visual cue condition, noVC = no visual cue condition. Bolded means indicate parametric analyses (ANOVA) were undertaken, bolded medians indicate the nonparametric tests Mann-Whitney and Wilcoxon Signed Rank tests were used. Details of statistical comparisons are presented in the text

#### Narrative Length

A two-way mixed ANOVA (group by condition) of the narrative word counts indicated a significant main effect of group (*F*(1,94) = 4.85, *p* = .030, η_p_^2^ = 0.049). The main effect of condition (*F*(1,94) = 3.47, *p* = .065, η_p_^2^ = 0.036) and the group by condition interaction (*F*(1,94) = 2.13, *p* = .148, η_p_^2^ = 0.022) were non-significant. Mean narrative length was shorter in the ADHD than NT group.

#### Narrative Structure

For the composite informativeness variable there was a significant group by condition interaction (*F*(1,94) = 6.59, *p* = .012, η_p_^2^ = 0.066). The main effects of group (*F*(1,94) = 15.78, *p* < .001, η_p_^2^ = 0.144) and condition (*F*(1,94) = 12.22, *p* < .001, η_p_^2^ = 0.115) were also significant. Children in the ADHD group produced less complete narratives than those in the NT group, this effect that was more pronounced in the no-cue condition. There was also a significant group by condition interaction for narrative coherence (global coherence rating) (*F*(1,94) = 6.86, *p* = .01, η_p_^2^ = 0.068) together with significant main effects for group (*F*(1,94) = 18.63, *p* < .001, η_p_^2^ = 0.165) and condition (*F*(1,94) = 54.77, *p* < .001, η_p_^2^ = 0.368). Coherence ratings were lower for the ADHD than the NT group, and in the no-cue than the visual cue condition, an effect that was larger for the ADHD group. Significant effects for informativeness and narrative coherence survived correction for multiple comparisons. Fig.S3 in Supplemental Materials presents the significant group by condition interactions for these two narrative variables. The interaction (*F*(1,94) = 0.18, *p* = .677, η_p_^2^ = 0.002), group (*F*(1,94) = 0.32, *p* = .576, η_p_^2^ = 0.003) and condition (*F*(1,95) = 0.18, *p* = .677, η_p_^2^ = 0.002) effects were non-significant for the inclusion of elaborations to engage the listener.

For off-topic deviations, group comparisons were carried out using the Mann-Whitney U test separately for each condition. These comparisons were non-significant for both the visual cue (*U* = 1027.00, *z* = -1.19, *p* = .233) and no-cue (*U* = 1001.50, *z* = -1.33, *p* = .185) conditions. Wilcoxon Signed Ranks Tests compared the effects of condition for each group separately. Off-topic deviations did not differ across conditions for the ADHD (*W* = 196.00, *z* = 1.45, *p* = .146) or NT groups (*W* = 55.00, *z* = 0.71, *p* = .475).

#### Narrative Fluency

There was a significant main effect of group (*F*(1,94) = 5.10, *p* = .026, η_p_^2^ = 0.051) for the log-transformed rate of self-interruptions. The main effect of condition (*F*(1,94) = 1.44, *p* = .233, η_p_^2^ = 0.015) and the group by condition interaction (*F*(1,94) = 0.42, *p* = .520, η_p_^2^ = 0.004) were non-significant. The narratives of the ADHD group included more self-interruptions than the NT group. For the log-transformed rate of pauses, the main effects of group (*F*(1,94) = 22.59, *p* < .001, η_p_^2^ = 0.194) and condition (*F*(1,94) = 6.64, *p* = .012, η_p_^2^ = 0.066) were significant, the group by condition interaction was not (*F*(1,94) = 0.00, *p* = .968, η_p_^2^ = 0.000). The rate of pauses was higher in the narratives of children with ADHD compared to the NT group and higher in the no-cue compared with the visual cue condition. The main effect of group was significant for the log-transformed rate of repetitions (*F*(1,94) = 4.45, *p* = .038, η_p_^2^ = 0.045). Neither the main effect of condition (*F*(1,94) = 2.32, *p* = .131, η_p_^2^ = 0.024) nor the group by condition interaction (*F*(1,94) = 0.86, *p* = .355, η_p_^2^ = 0.009) were significant. Children in the ADHD group evidenced a higher rate of repetitions than the NT group. For the log-transformed rate of fillers, there were no significant effects for group (*F*(1,94) = 3.26, *p* = .074, η_p_^2^ = 0.034) or condition (*F*(1,94) = 3.89, *p* = .052, η_p_^2^ = 0.034), and the group by condition interaction (F(1,94) = 0.56, *p* = .355, η_p_^2^ = 0.009) was non-significant. The significant group effect for pauses was the only comparison to survive correction for multiple comparisons.

Chi-square was used to compare the proportion of false starts between the ADHD and NT groups separately for each condition. No significant group differences were found for the visual cue (χ^2^(1, *N* = 96) = 0.7, *p* = .793) or no-cue conditions (χ^2^(1, *N* = 96) = 2.67, *p* = .102); the likelihood of making false starts did not differ across groups in either condition. McNemar’s test was used to compare the number of false starts across conditions. Overall, children made more false starts in the no-cue than in the cue condition (χ²(1, *N* = 96) = 7.85, *p* = .005). When analyzed separately, this effect was significant for the ADHD group (χ²(1, *n* = 50) = 7.26, *p* = .007), but not the NT group (χ²(1, *n* = 46) = 0.84, *p* = .359).

#### Linguistic Accuracy

For the log-transformed rate of referencing errors, there was a significant main effect of group (*F*(1,94) = 4.86, *p =* .03, η_p_^2^ = 0.049). The condition effect (*F*(1,94) = 1.65, *p* = .203, η_p_^2^ = 0.017) and the group by condition interaction (*F*(1,94) = 3.72, *p* = .057, η_p_^2^ = 0.038) were non-significant. Children in the ADHD group produced higher rates of referencing errors than NT children. The main effects of group (*F*(1,94) = 27.30, *p* < .001, η_p_^2^ = 0.225) and condition (*F*(1,94) = 4.91, *p* = .029, η_p_^2^ = 0.050) were significant for the log-transformed rated of cohesion errors. The group by condition interaction (*F*(1,94) = 1.51, *p* = .223, η_p_^2^ = 0.016) was not significant. The mean rate of cohesion errors was higher in the ADHD group than the NT group, and in the no-cue than the visual cue condition. For the log-transformed rates of semantic and morphosyntactic errors (combined), the main effects of group (*F*(1,94) = 41.17, *p* < .001, η_p_^2^ = 0.305) and condition (*F*(1,94) = 4.96, *p* = .028, η_p_^2^ = 0.050) were significant. The group by condition interaction (*F*(1,94) = 3.22, *p* = .076, η_p_^2^ = 0.033) was not significant. Children in the ADHD group had significantly higher error rates compared to NT children. Error rates were higher in the no-cue than the visual cue condition. Only the significant group effects for cohesion errors and semantic and grammar errors survived correction for multiple comparisons.

Phonological error rates were analyzed using nonparametric statistics. The groups were compared on this variable separately for the two conditions using the Mann-Whitney U test. Children in the ADHD group showed significantly higher rates of phonological errors than the NT group in the visual cue condition (*U* = 927.00, *z* = − 2.66, *p* = .008). Error rates were not significantly different across groups in the no-cue condition (*U* = 1087.00, *z* = − 0.092, *p* = .360). Within-group comparisons of the effect of condition, using the Wilcoxon Signed Ranks Test, showed no significant differences between conditions in the ADHD (*W* = 51.00, *z* = -1.64, *p* = .101) or NT (*W* = 55.00, *z* = 0.71, *p* = .475) groups.

#### Exploratory Analyses

Hierarchical multiple regression analyses were carried with informativeness, narrative coherence, pauses, cohesion errors, and semantic and grammar errors as the outcome variables. Two models were tested, in the first model symptom severity was entered at step 1, working memory as step 2, and core language at step 3. In the second model the order of entry of working memory and core language was reversed. Table 6 presents the percentage of variance explained for each model separately by condition.

**Table 6:** Percentage of variance in narrative performance explained by (variance in) ADHD symptom severity, working memory and core language skills in the hierarchical regression models for the two conditions

VC condition						
Outcome variables		Model 1		Model 2		
	ADHD step 1	WM step 2	Language step 3	Language step 2	WM step 3	
Informativeness	1.9%	1.5%	**4.8%**	**6.2%**	0.1%	
Coherence	3.5%	1.9%	**4.4%**	**6.2%**	0.0%	
Pauses	**6.0%**	**6.5%**	2.4%	**7.8%**	1.1%	
Cohesion errors	**7.0%**	0.3%	**5.6%**	**4.9%**	1.0%	
Semantic and grammar errors	**19.9%**	1.6%	2.8%	**4.4%**	0.0%	
noVC condition						
Outcome variables		Model 1		Model 2		
	ADHD step 1	WM step 2	Language step 3	Language step 2	WM step 3	
Informativeness	**9.5%**	0.0%	0.6%	0.3%	0.3%	
Coherence	**11.0%**	**3.8%**	3.2%	**6.7%**	0.2%	
Pauses	3.5%	1.4%	0.1%	0.3%	1.2%	
Cohesion errors	**9.0%**	**6.8%**	0.7%	**5.1%**	2.4%	
Semantic and grammar errors	**17.4%**	**5.9%**	**8.7%**	**14.6%**	0.0%	

VC = visual cue, noVC = no visual cue, WM = working memory. ADHD symptom severity was entered as step 1 in both models. Significant findings are presented in bold

In the visual cue condition, entered at step 2, working memory explained significant variance over and above ADHD severity for pauses only. Entered after working memory, core language skills explained significant additional variance in informativeness, narrative coherence, and cohesion errors. Entered ahead of working memory, language scores explained significant variance in all the outcome variables, with no additional variance explained by working memory. A different pattern emerged for the no visual cue condition. At step 2, working memory explained significant additional variance in narrative coherence, cohesion errors and semantic and grammar errors, but not pauses. Entered after working memory language explained additional significant variance in semantic and grammar errors only. Entered ahead of working memory, language explained significant additional variance in narrative coherence, cohesion errors and semantic and grammar errors, with no additional significant variance explained by working memory. In the no cue condition neither working memory of nor language explained significant additional variance in informativeness or pauses.

## Discussion

The current study compared the narrative skills of children with and without ADHD in a story retelling task under different working memory loads, i.e., with and without visual story cues. As a group, children with ADHD produced shorter stories that had weaker narrative structure and were less fluent and accurate than their NT peers. The presentation of story pictures improved performance in both groups.

Children with ADHD produced stories that were less informative and less coherent than those of their peers. The size of these effects, their presence across conditions, and the consistency of similar findings in the literature, suggest impairments in these skills in children with ADHD (e.g., Bergman & Hallin, 2021; Freer et al., 2011; Lorch et al., 2010; Purvis & Tannock, 1997). The absence of group differences for the other narrative structure variables (off-topic deviations and the presence of elaborations) may reflect the brevity of the target stories and/or the structured nature of the task, i.e., inclusion of specific instructions, and the predetermined story topics and events.

The narratives of the children with ADHD were less fluent than those of their peers. While earlier studies report more dysfluencies in the narratives of children with ADHD, they provide limited information on specific dysfluencies (Kuijper et al., 2017; Lee et al., 2017). Here we compared dysfluency types separately, with implications for understanding and treating narrative fluency difficulties in children with ADHD. Rates of self-interruptions, repetitions, and pauses were all significantly higher in the ADHD group. The largest effect was for pauses, perhaps reflecting a breakdown in planning in those with ADHD. Although the effects were smaller, group differences in self-interruptions and repetitions suggest monitoring or word-finding weaknesses. These latter effects did not survive correction for multiple comparisons. The overall pattern of findings suggest the core weakness in narrative fluency may be in planning and organizing utterances.

Linguistic accuracy was lower in the ADHD group than the NT group. The narratives of children with ADHD included more errors in referencing, cohesion, semantics and morphosyntax, and phonology. Except for phonological errors, these differences are reported in the literature, but not within the same study (Bergman & Hallin, 2021; Kuijper et al., 2015, 2017; Lorch et al., 1999; Purvis & Tannock, 1997; Tannock et al., 1993). The presence of these errors, in the same sample of children, indicate linguistic inaccuracies span multiple levels of processing, i.e., semantic content and grammatical relationships (word/sentence level) as well as speech planning and phonological encoding (phoneme/sound level), with implications for assessment and intervention in children with ADHD.

The number and range of variables included in the current study is larger than in any previous study and highlights the extent of the narrative difficulties experienced by children with ADHD. The breadth of difficulties observed, suggests that narrative production in ADHD is not disrupted at a single stage of processing, but rather across multiple levels of the speech production system.

We predicted the presence of visual cues would improve performance in the ADHD group, possibly through reducing the (working memory) load imposed by the task. Predictions regarding the effects of story cues on NT group performance were more circumspect. The addition of visual cues improved performance, *across groups*, on several variables. The children’s stories were more complete and coherent in the visual cue condition, an effect that was more pronounced for children with ADHD. The presence of visual cues also improved aspects of narrative fluency and linguistic accuracy. The rate of pauses and the proportion of children who made false starts (ADHD group only) was lower in the visual cue condition. Cohesion, semantic, and morphosyntactic errors rates were also lower when visual cues were present. Only the condition effects for the narrative structure variables of informativeness and coherence survived correction for multiple comparisons.

These condition effects offer some support for the hypothesis that visual cues improve story retelling in children with ADHD. However, improvements were not found for all of narrative variables assessed, and the presence of visual cues did not normalize the performance of the children with ADHD. Consistent with the findings of Bergman and Hallin (2021), NT children also benefited from the presentation of story pictures, with differential condition effects found only for informativeness and narrative coherence. For both variables, children with ADHD benefited more than NT children from the inclusion of visual cues. The smaller impact of cues on the narratives of the NT group might reflect ceiling effects. From an interventional perspective, visual prompts are expected to improve children’s narrative structure and may support their fluency and linguistic accuracy.

In the introduction, we suggested ADHD symptoms and lower language and working memory skills might contribute to poorer narrative performance in children with ADHD. In the current study, mean working memory and general language scores were significantly lower in the ADHD than NT group, but showed only small to moderate associations with the narrative variables. Group membership was based on the number and severity of ADHD symptoms. The extent, i.e., number and size, of the group differences in narrative performance may reflect the additive effects of ADHD symptoms, working memory and general language skills. Visual cue effects were larger in the ADHD group for the narrative structure variables, suggesting recall and organization of story content may rely more on working memory than the other narrative variables. Reducing cognitive demand (i.e., intrinsic load) through providing sequential visual cues would be expected to produce greater gains in those with reduced working memory capacity, including individuals with ADHD.

The exploratory regression analyses offer some insight into the contributions of ADHD, working memory and core language skills to narrative performance across study participants. During unaided retelling, symptom severity explained significant variance in narrative structure and linguistic accuracy, with working memory explaining significant additional variance. Core language explained additional variance in linguistic accuracy only. These findings offer some evidence for the additive contribution of these variables to narrative performance.

The pattern of associations differed when visual story cues were presented. ADHD severity continued to explain variance in linguistic accuracy but not narrative structure. The ordered cues likely provided the necessary (external) structure, but did not mitigate the effects of inattention and impulsivity/hyperactivity on linguistic accuracy. Not surprisingly, when cognitive load was reduced, working memory no longer explained significant variance in narrative structure or linguistic accuracy. In this condition core language was the only variable to explain significant variance in narrative structure, highlighting the interplay between structural language skills and language use in discourse.

### Strengths and Limitations

The current study has several important strengths. It is the first study to directly test the effects of visual cues on story retelling in pre-adolescent children with ADHD. It coded for a broad range of narrative variables within a single study, offering a comprehensive profile of the children’s narrative skills. The study methodology is sound, the diagnostic practices were thorough, the stories well matched with presentation order and condition counterbalanced, the coding scheme reliable, and the raters blind to group membership and other child characteristics. As a volunteer sample, rates of comorbidity in the ADHD group were low. This increases the generalizability of the findings to the broader community of children with ADHD. However, it limits our understanding of the impact of co-occurring conditions on the narrative skills of children with ADHD.

There are some study limitations. It is possible that the story pictures themselves introduced a visual cognitive load. However, this seems unlikely given performance was better in the visual cue condition across groups. The stories were relatively short with a simple structure compared to those used in some other studies (e.g., Bergman & Hallin, 2021; Kuijper et al., 2017; Rumpf et al., 2012), possibly leading to ceiling effects on some variables. The bespoke task is not normed, preventing determination of impairment in narrative skills. Standardized narrative measures were not included in the current study, preventing comparisons with the study specific narrative coding scheme. Additional research is needed to formally establish the validity of the coding scheme. Group comparisons of intellectual functioning were based on Estimated SFIQ scores. Such short forms magnify the effects of administrative errors and place greater weight on the included subtests than long forms (Sattler, 2024).

## Conclusion

The current study adds to our understanding of the narrative skills profile of children with ADHD, including the impact of memory load on story retelling. Group differences were identified on measures of narrative structure, fluency and accuracy, suggesting children with ADHD have greater difficulty in planning and monitoring speech output than their peers. The inclusion of visual cues improved story retelling performance across groups, with larger effects for the ADHD group on aspects of narrative structure, but it did not normalize their performance. ADHD characteristics, working memory and general language skills contribute to children’s story retelling, their importance varying for different aspects of narrative performance and with the presence of visual cues.

Given the importance of storytelling to children’s successful communication and social interactions, the current findings argue for including narrative skills in language assessments for children with ADHD. This should include evaluation of narrative structure, fluency and linguistic accuracy. Extrapolating beyond the current findings, targeted narrative language interventions should focus on enhancing story organization, coherence and linguistic precision, as well as fluency. The heterogeneity of ADHD suggests that narrative assessments and interventions should not be one-size-fits-all. Children with different symptom profiles or co-occurring language impairments may require different targets for intervention. Given the positive impact of visual cues on story retelling, clinicians, educators, and caregivers should consider providing such prompts to encourage linguistic organization.

## Supplementary Information

Below is the link to the electronic supplementary material.

Supplementary Material 1

## Footnotes

1 See Supplemental Materials for a glossary of relevant narrative and linguistic terms.

2 Cognitive Load Theory divides cognitive load into three categories, intrinsic (task difficulty), extraneous (unnecessary information or activities) and germane (mental effort required for deep processing) that compete for available working memory capacity.

3 Six or more symptoms of inattention and/or hyperactivity/impulsivity in at least one setting and evidence of symptoms in a second setting (i.e., home, school, or clinic), impairment from symptoms.

4 Sattler reports that “short forms of the WISC-V may be used (i) for screening, (ii) research, or (iii) to obtain an estimate of a child’s intellectual status when a precise FSIQ is not required.” (p. 263). Short forms with reliabilities and validities *≥* 0.80 can be used with confidence (Sattler, 2024).

5 This included children with CELF-5 Core Language Scores (CLS) < 80 (Wiig et al., 2013).

6 The CBRS and Conners 3 forms demonstrate high levels of internal consistency, temporal stability and inter-rater reliability, see Table S1 Supplemental Materials.

7 K-SADS reported inter-rater reliability coefficients range from 0.77 to 0.97 for the ADHD section, 0.85 to 0.96 for the ODD section, and 0.78 to 1.0 for the CD section (de la Peña et al., 2018; Makino et al., 2023; Nishiyama et al., 2020).

8 All research team members who worked directly with the children completed detailed written observations of their presentation/behavior during interviews and testing.

9 SFIQ scores were calculated for all participants to allow for group comparisons of estimated SFIQ.

10 One participant in the NT group did not complete the CELF-5 due to time constraints.

11 Children in the ADHD group and most children in the NT group were administered the narrative task during the morning.

12 As very few event sequencing errors occurred, this variable was not included in data analysis.

13 The model (k,3) was used, as it calculates raters’ ranking consistency while factoring in their variance and distinguishes between systematic rater bias versus random disagreements.

14 CELF-5 scores for boys and girls were not significantly different in either the ADHD or NT groups. ADHD boys *M* = 100.22, *SD* = 11.56, girls *M* = 99.43, *SD* = 13.17, *t*(48) = 0.225, *p* = .823; NT boys *M* = 106.83, *SD* = 9.47, girls *M* = 109.63, SD = 12.09, *t*(43) = − 0.826, *p* = .414.
