# Supplementary material for: A Picture is Worth How Many Words? The Impact of Visual Cues on the Story Retelling of Children with and without ADHD
Source: Res Child Adolesc Psychopathol. 2026 Aug 20;54(5):100. doi: 10.1007/s10802-026-01494-3 (PMC13493489; doi:10.1007/s10802-026-01494-3)
Supplement: Supplementary file 1 [file 10802_2026_1494_MOESM1_ESM.pdf]

Supplemental Materials

Glossary for Narrative/Linguistic Terms

Narrative Completion: The degree to which a story includes a proper structure and necessary facts.

Plot Units: The essential functional components of a story (e.g., initiating event, the character's internal response, the attempt, the consequence).

Story Grammar Units: The mental schema the storyteller uses to understand and construct a story (e.g., setting, initiating event, internal response, plan, attempt, direct outcome, reaction).

Topic Maintenance: The speaker's ability to stay on the central theme of the narrative without introducing irrelevant digressions.

Event Sequencing: The chronological ordering of story events in a logical temporal sequence to reflect the story's timeline.

Explicitness: The clarity and completeness of information provided to understand the story without needing external context.

Fluency: The smooth, uninterrupted flow of speech during narration.

Morphosyntax: The combined domain of grammar, encompassing word structure (morphology) and sentence structure (syntax) within utterances.

Morphosyntactic Complexity: The complexity of sentence structuring, including the use of subordinate clauses and phrases.

Cohesion: The use of cohesive devices (e.g., conjunctions) to connect words, clauses or sentences.

Referencing: The clear introduction and subsequent mention of characters and objects, typically using full nouns first and clear pronouns thereafter.

Semantics: The domain of meaning, including word choice, vocabulary, and the accurate conveyance of ideas and relationships within the story.

Table S1

Reliability for the Conners Behavior Rating Scales (CBRS) and the Conners 3

Form		Internal consistency[1] (α)	Test-retest (*r*)	Inter-rater (*r*)
CBRS-parent	Content scales	.78-.95	.70-.96	.62-.89
	DSM-IV symptom scales	.73-.93	.66-.95	.53-.84
CBRS-teacher	Content scales	.81-.97	.80-.96	.50-.88
	DSM-IV symptom scales	.69-.95	.76-.94	.53-.98
Conners-3-parent	Content scale	.85-.94	.72-.98	.74-.84
	DSM-IV symptom scales	.83-.93	.84-.94	.75-.94

Note. data extracted from Conners (2008a) and Conners (2008b).

[1] = Chronbach's alpha

Table S2

Details of Story Length and Complexity

	Story	
Variable	The Greedy Dog	The Bald Man and the Fly
Word count	91	83
Narration time	55 seconds	52 seconds
Character count	2	2
Plot unit count	4	4

Fig.S1

Images Depicting the Story "The Greedy Dog"

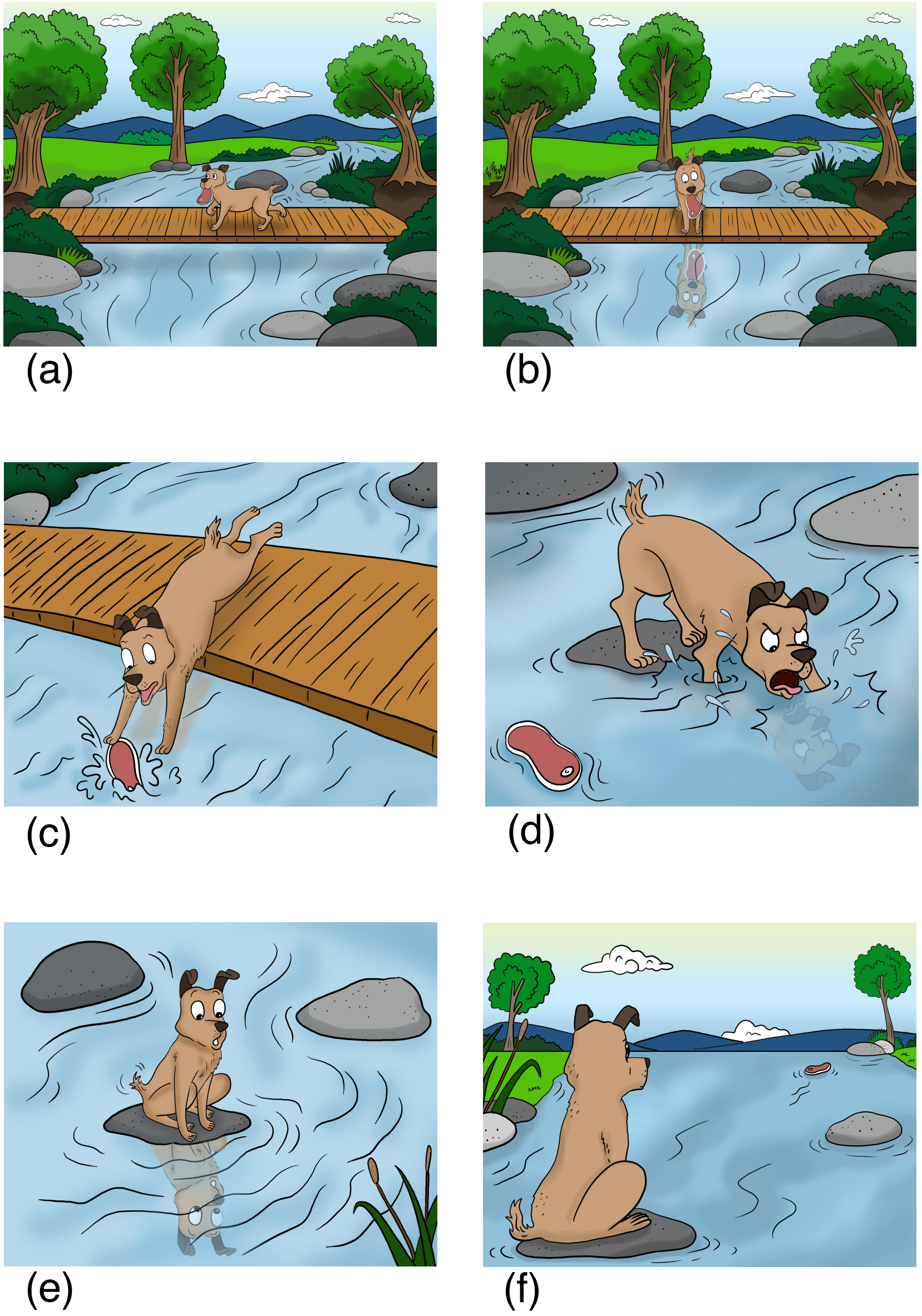

Fig.S2

Images Depicting the Story "The Bald Man and the Fly"

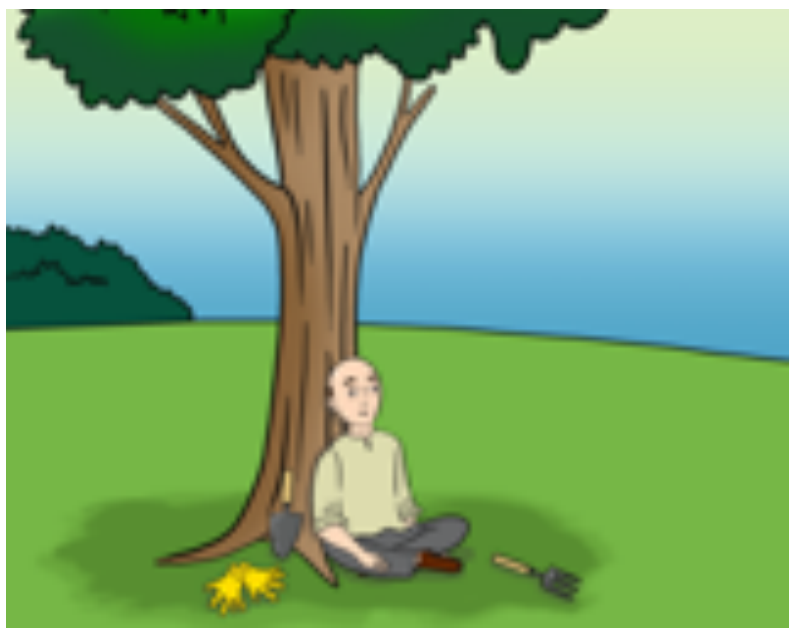

(a)

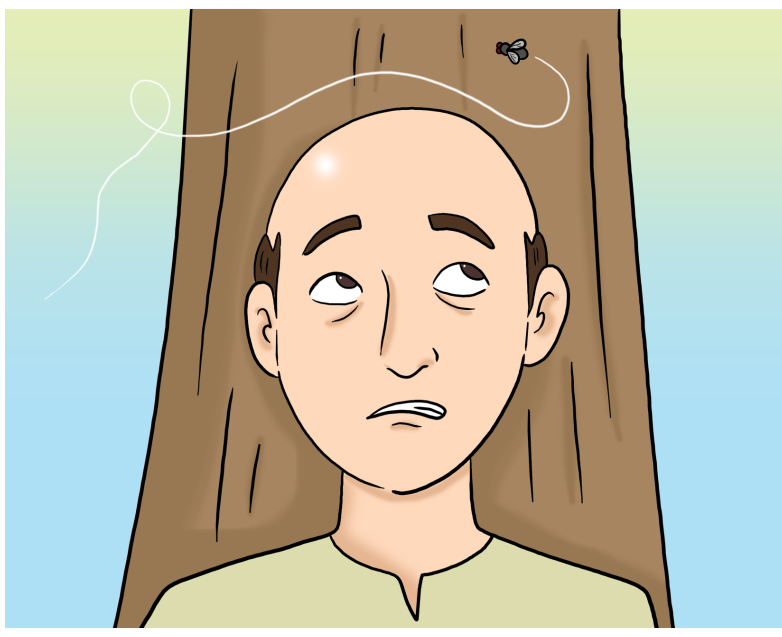

(b)

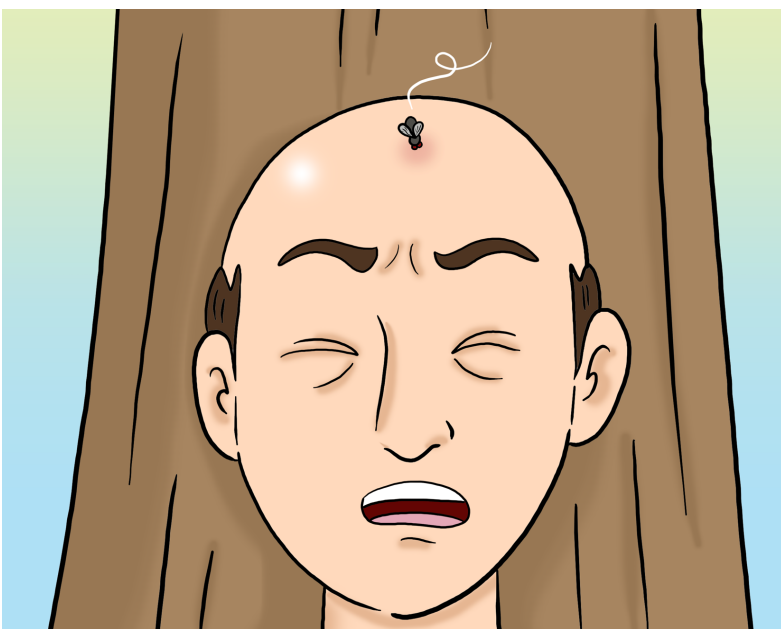

(c)

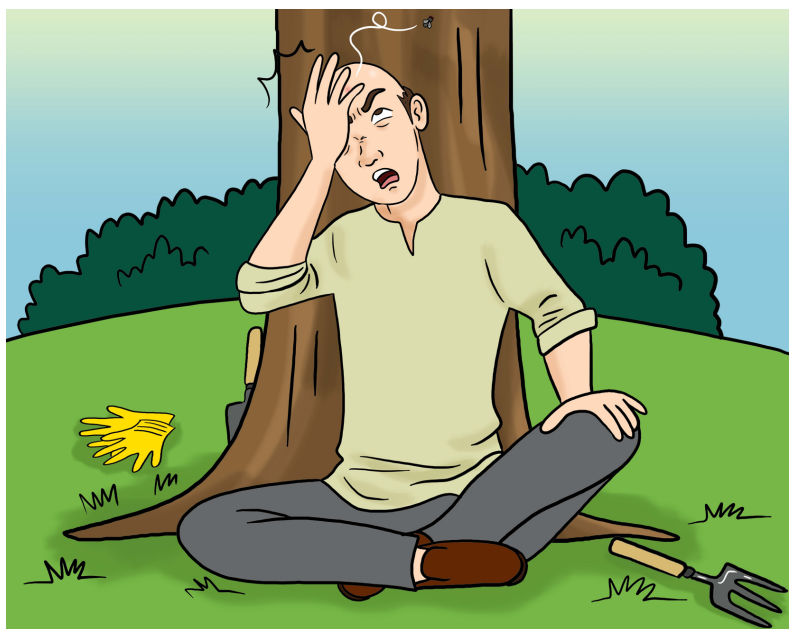

(d)

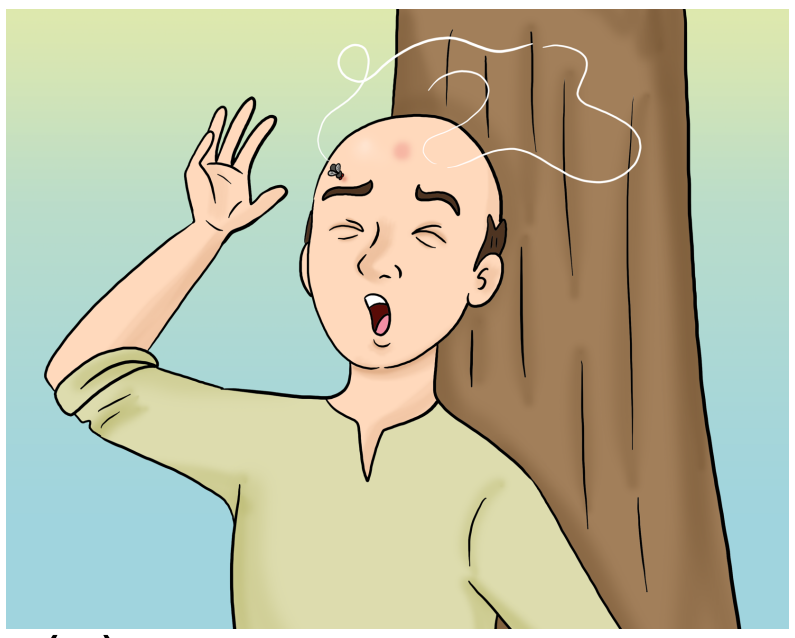

(e)

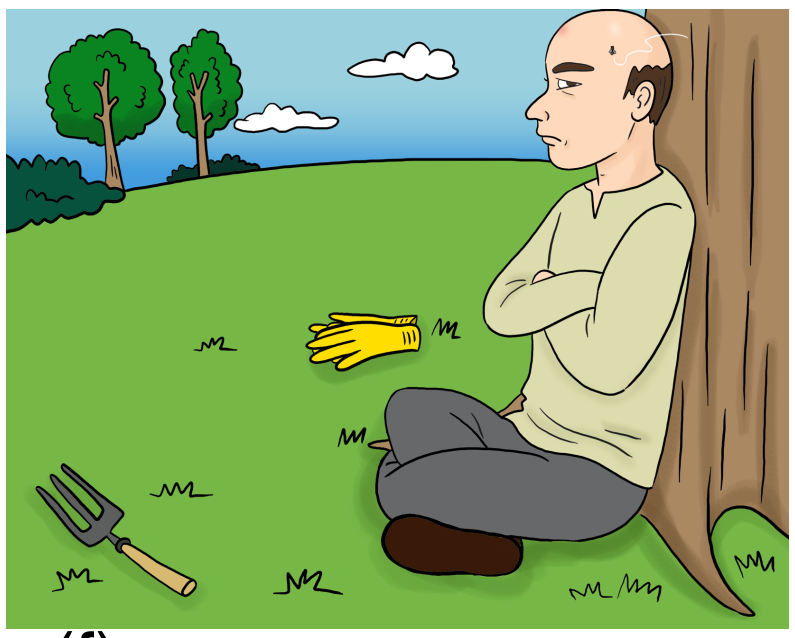

(f)

Task Protocol and Participant Instructions

Below is the narrative task protocol. The parts in blue were said out loud to the participant.

In the current study the order of presentation of the visual-cue and no visual cue conditions were counterbalanced across participants.

General instructions:

Child receives task specific instructions. “You will listen to two stories and be asked to repeat them afterwards while pretending to be a news reporter, a camera will record you so you can watch yourself on the screen after we’re done”.

Visual-cues condition:

Child receives specific instructions for visual-cue condition. “You will watch this story on the screen with pictures and a voice, and then repeat it while watching the same pictures but without the voice - you will replace the voice. Try to remember the story as best as you can and repeat it as accurately as you can”.

1. Child listens to the narration with the pictures on a screen.

2. Child is reminded that they will retell the story while watching the pictures. “Try to remember the story as best as you can, and repeat it as accurately as you can”

3. (Camera recording) Child retells the story. The experimenter will control the slide transition according to the child’s narration. If child is struggling to speak, the experimenter will say “you can say anything you remember”.

Child is given a 3-minute break before the second story.

No-cue condition:

Child receives instructions for no-cue condition “You will watch another story, after it’s over, you will retell it while there is a blank screen on the monitor. Try to remember the story as best as you can, and repeat it as accurately as you can”

1. Child listens to the narration with the pictures on a screen.

2. Blank screen remains on for whole narration. Child is reminded that they will retell the story. “Try to remember the story as best as you can, and repeat it as accurately as you can”

3. (Camera recording) Child retells the story. If child is struggling to speak the experimenter will “you can say anything you remember”.

Narrative Transcribing Guidelines

Below are guidelines and instructions for transcribing an audio recording of a child telling/retelling a story. These guidelines are not specific to the current study.

The topic of each guideline is in an underlined blue text.

Some anonymized examples of children speaking are presented; they are typed in red *italics* with quotation marks "……".

Content of transcription

Type all utterances of the participants verbatim, including:

1. Meaningless words, meaningless syllables, single vowels or consonants. Type these in square brackets [], according to the phonetic alphabet rules.

   Example

   *"……**[d]** who do what?"*

   The child started saying something *"[d]" that had the sound of the consonant /d/* but then revised to *"who"*

2. Partial repetitions of words
   Even though these are word fragments/partial words, there is no need to type them in accordance with the phonetic alphabet rules since we already know what the intended word is. Type the part of the word that is audible.

   Example

   *"... maybe [ne] she could say like maybe next time **ime** you could show me but I really wanna get some pocket money"*

   the speaker partially repeated the word *"time"* - *"ime"*.

3. Full word repetitions
   When a speaker repeats a word, that repetition should be typed into the transcript.

   Example

   *"Because she [t] she won't know **if if** it if she means by um……um……"*

   The speaker repeated *"if"* twice within the same utterance

4. Filler words i.e., um, uh, eh, hm
   All filler words should be included in the transcript.

   Example

   "Because she [t] she won't know if if it if she means by ***um……um……"***

5. Any type of linguistic errors, even if revised (for example grammatical or vocabulary errors)

   Example

   "the dog **were** was walking"

Pauses and silence

1. A pause is when a speaker pauses mid-sentence.

   -Indicate a short pause or short silence that lasts for 2-3 seconds with three dots "…"

   - indicate a long pause or long silence period that lasts more than 3 seconds with six dots "……"

   Example

   um she could say [meyb] maybe… maybe we could play together so you so you [a] show me another time"

   There is a short pause (2-3 seconds) after the word "*maybe"*

2. Long pause between sentences. Type (pause) if the was a pause of 5 seconds or more between sentences.

   Example

   Once there was a dog walking on a bridge with a big piece of meat in his mouth. (pause) He looks down at the water and saw his reflection.

Questions

Type a question mark at the end of every utterance that:

1. Has the clear structure of a question.

2. Other utterances that don't have a structure of a question, but a clear ascending intonation of a question (upwards swing). In this case type (question intonation) right after the question mark.

   Example

   "To say…see you next time (question intonation)"

   The speaker's utterance doesn't have a question structure, but the intonation was one of a question, i.e., the speaker was offering an answer but was wondering if it was correct as evidenced by raising intonation.

Elongated sounds

When the speaker pronounces elongated (stressed) syllables or vowels, type the elongated letters in **bold**. And type (elongated sound) after the word that contains the sound.

Example

*"there was a d**o**g" (elongated sound)*

Inaudible utterances

1. Type (inaudible) in parentheses to indicate inaudible or unclear words that are muffled due to bad audio quality, i.e., the microphone or person moved.

2. Type (extended inaudible) to indicate extended inaudible utterances.

Unclear speech

Type the phrase (unclear speech) in parentheses if the pronunciation of the speaker is unclear in one word and it's not possible to infer the word.

Type (extended unclear speech) if the pronunciation of the speaker is unclear for more than one word.

Other verbalizations

Type in parentheses () other actions other than talking that can be inferred reliably from the audio e.g., (clears throat), (sings), (mumbles), (vocal noise) i.e., prolonged vocalizations that have no meaning

Narrative Coding Scheme and Manual

These guidelines are for coding several elements found in storytelling.

You are asked to code transcripts of stories told by children. Each transcript includes two stories, the stories are presented below:

Story 1- “The dog and the shadow”:

> A dog, was crossing a bridge over a stream with a piece of meat in his mouth. He saw his own shadow in the water and thought it was another dog, with a piece of meat twice as big as his. He immediately let go of his own piece of meat, and fiercely attacked the other Dog to get his bigger piece. As a result, he lost both: the one he tried to catch in the water, because it was a shadow; and his own, because the stream swept it away.

Story 2- “The bald man and the fly”:

> On a hot summer’s day, a bald man sat down to rest after a long day of work. A fly came up and kept buzzing around his bald head. Then the fly stung him. The man aimed to smack the little fly, but his palm hit his head instead. Again, the fly kept flying around his bald head and stinging it. But this time, the man decided not to try and smack the fly, because he didn’t want to slap himself instead.

The storytelling elements you are asked to code are:

1. Topic maintenance
2. Event sequencing
3. Explicitness and coherence
4. Referencing
5. Conjunctive cohesion
6. Fluency
7. Semantic mistakes
8. Phonological mistakes
9. Morphosyntactic mistakes

Within each element there are one or more variables that will be coded. You will code from transcripts of stories told by the participants, and from the scripts of the original stories. You are asked to mark the occurrence or absence of each variable in the

transcript; the occurrences are marked on the transcript, and the absences are marked on the scripts of the original stories.

The variables you are asked to code are elaborated below, with examples typed in blue:

Element 1- topic maintenance

-Deviation off topic: this includes any adding extraneous events, stating irrelevant or false likes/dislikes/capabilities/feeling of characters, adding false, irrelevant, or ambiguous details. Mark the occurrences of each deviation off topic with the code DOT.

Example:

The fly kept buzzing and then it bit his bald head. And then he tried to slap it and hit his head instead and then… it went away again and then it did it again".

In the example above, the highlighted part is a false detail in which the is fly going away again.

Element 2- event sequencing

The main events and the right sequence of each story are presented below:

Story 1- 1. Dog crossed bridge while holding a piece of meat in his mouth. 2. He saw his own shadow. 3. He let go of the meat 4. He attacked the shadow. 5. The stream swept the meat away (he lost the meat).

Story 2- 1. A man sat to rest after work. 2. A fly buzzed around his head. 3. The fly stung him. 4. The man smacked himself 5. The fly kept buzzing around his head, but he did nothing.

-Mark each achronological presentation of an event with the code AP.

Example of achronological presentation:

“he saw his own reflection and thought it was another dog holding a bigger piece of meat two times as big and he…and he went into the water and let go of his piece of meat”.

In the example above, the participant presented the event where the dog goes into the water to attack the shadow before the event where he dropped the meat.

-Mark with the code OE each missing event on the actual event in the original story script.

Example of missing event:

“he saw another dog with meat twice, his meat was twice as big as his… so he jumped in to go and fight him for the meat…and he realized……it was him”

In the story above, the event in which the dog let go of his meat is missing.

Element 3- explicitness and coherence

-Introduction of all characters and objects.

The characters and objects that should be introduced include:

Story 1- Main character: Dog

Objects or minor characters: Other dog/shadow, Meat, bridge

Story 2- Main character: Man

Objects or minor characters: Fly, bald head, palm

Mark each missing character/object with the code MC on the original story script.

Example of missing character or object:

"The fly stung him but the man hit himself, the fly kept stinging him".

In the example above, the participant failed to mention the object "bald head".

-Delivery of all basic facts.

The basic facts of each story are presented below:

Story 1- 1. There was a dog crossing a bridge holding meat. 2. He saw his shadow in the water 3. He let go of his own meat and attacked the other dog to get his meat. 4. He lost his meat

Story 2- 1. A man sat to rest after work. 2. A fly stung his head. 3. He aimed to smack the fly but smacked himself instead. 4. The fly kept buzzing around his head.

Mark each missing or wrong basic fact with the code BF on the original story script.

Example of missing fact:

"A bald man was sitting by a tree after a long day of work. And then a fly came and kept on buzzing around his bald head. And then he tried to smack it! But he hit his bald head".

In the example above, the participant omitted the fact that the fly stung the man before he tried to smack it.

-Presence of elaboration to engage the listener

Examples of elaborations that are considered additional details beyond the basic facts are typed in green as presented below:

Story 1- A dog, was crossing a bridge over a stream with a piece of meat in his mouth. He saw his own shadow in the water and thought it was another dog, with a piece of meat twice as big as his. He immediately let go of his own piece of meat, and fiercely attacked the other Dog to get his bigger piece. As a result, he lost both: the one he tried to catch in the water, because it was a shadow; and his own, because the stream swept it away.

Story 2- On a hot summer's day, a bald man sat down to rest after a long day of work. A fly came up and kept buzzing around his bald head. Then the fly stung him. The man aimed a to smack the little fly, but his palm hit his head instead. Again, the fly kept flying around his bald head and stinging it. But this time, the man decided not to try and smack the fly, because he didn't want to slap himself instead.

Mark the presence of each elaboration with the code E.

Example of elaboration:

"The dog was walking across the bridge. In the water he saw his own reflection he jumped in because he saw a bigger piece of meat in a dog's mouth then then he jumped and then he lost both meats, the one from the shadow and the real one that he had. And then it was swiped away down the stream"

In the example above, the highlighted details are two of the additional details mentioned in the original story.

-Completion of story

The presence of all plot units indicates completion of story. The full stories are presented below while separated into units (Introduction, problem, action, and realization/conclusion):

Story 1- "The dog and the shadow":

Introduction: "A dog, was crossing a bridge over a stream with a piece of meat in his mouth"

Problem: “He saw his own shadow in the water and thought it was another dog, with a piece of meat twice as big as his”

Action (attempt to solve the problem): “He immediately let go of his own piece of meat, and fiercely attacked the other Dog to get his bigger piece”

Realization/conclusion: “As a result, he lost both: the one he tried to catch in the water, because it was a shadow; and his own, because the stream swept it away”

Story 2- “The bald man and the fly”:

Introduction: “On a hot summer’s day, a Bald man sat down to rest after a long day of work”

Problem: “A Fly came up and kept buzzing around his bald head. Then the fly stung him”

Action (attempt to solve the problem): “The Man aimed a to smack the little fly, but his palm hit his head instead”

Realization/conclusion: “Again, the Fly kept flying around his bald head and stinging it. But this time, the man decided not to try and smack the fly, because he didn’t want to slap himself instead.”

Mark each missing or inadequate unit as MU on the original story script.

Example of missing or inadequate unit:

“The dog was walking across the bridge. In the water he saw his own reflection he jumped and he lost both meats, the one from the shadow and the real one that he had”.

In the story above, in the “action” unit, the participant only mentions that the dog jumped, this is considered inadequate unit

since it does not include most of the details.

-Global coherence

Rate the overall coherence of the story from 1 to 5. Take into consideration the logical and thematic unity of the entire narrative.

Element 4- referencing

Below the proper referencing while introducing the characters and objects are highlights in each story:

Story 1- *A dog,* was crossing a *bridge* over a stream with *a piece of meat* in his mouth. He saw his own shadow in the water and thought it was *another dog*, with a piece of meat twice as big as his. He immediately let go of his own piece of meat, and fiercely attacked the other Dog to get his bigger piece. As a result, he lost both: "the one" "he" tried to catch in the water, because it was a shadow; and his own, because the stream swept it away.

Story 2- On a hot summer's day, *a Bald man* sat down to rest after a long day of work. *A Fly* came up and kept buzzing around his bald *head*. Then the fly stung him. The Man aimed a to smack the little fly, but his *palm* hit his head instead. Again, the Fly kept flying around his bald head and stinging it. But this time, the man decided not to try and smack the fly, because he didn't want to slap himself instead.

-Using pronouns or vague introduction with no prior introduction characters and objects; or the wrong pronoun or wrong referencing: Mark with RE for any referencing error. "Shadow" in story 1 can be replaced with "reflection".

Examples of failure of introduction:

"After a long day of work, the man decided to go under a tree and and rest".

In this example, the participant did not introduce the "bald man", they went on using a vague referencing using "the man". Using "the" gives the impression that the character was already introduced, but in this example, it's being mentioned for the first time, therefore making it a referencing error.

Example of wrong referencing:

"The fly was buzzing around, and he stung him".

The participant referred to the fly as "he" which is a wrong pronoun.

Note: the one highlighted in blue in story 1 Can be substituted for other synonyms like "the piece", "the meat".

Element 5- Conjunctive cohesion

The use of words and phrases that link utterances and events (temporal links, coordination links, causality, enabling, disjunction). The words typed in blue below are examples of cohesive devices:

Story 1- A dog, was crossing a bridge over a stream with a piece of meat in his mouth. He saw his own shadow in the water and thought it was another dog, with a piece of meat twice as big as his. He immediately let go of his own piece of meat, and fiercely attacked the other Dog to get his bigger piece. As a result, he lost both: the one he tried to catch in the water, because it was a shadow; and his own, because the stream swept it away.

Story 2- On a hot summer's day, a bald man sat down to rest after a long day of work. A fly came up and kept buzzing around his bald head. Then the fly stung him.

The man aimed to smack the little fly, but his palm hit his head instead. Again, the fly kept flying around his bald head and stinging it. But this time, the man decided not to try and smack the fly, because he didn't want to slap himself instead.

- The cohesive devices children use will be highlighted in the actual transcripts. Mark any cohesion errors (Missing or using the wrong device) with the code C.

Example of error in using cohesive devices:

"The dog was walking across the bridge with meat in his mouth but saw another dog with bigger meat. He accidentally dropped the meat out of his mouth and then he tried to fight after the dog and then he lost his meat".

In the story above, the word but incorrectly implies a contrast between the action of walking with the meat and the act of seeing the other dog, when the relationship is one of simple sequence. The second highlighted cohesive device is an error as well because the dog lost the meat as a result of dropping it before attacking the dog, and not after he attacked the dog.

Element 6- Fluency

-False starts:

Any false start of the story that is retracted and then the speaker starts over or corrects themselves. This refers only to the introduction of the story. Mark any false start with the code FS.

Example of false start:

"There was a man on a hot sunny day, under a tree sitting down- a bald man I mean, sitting under a tree".

In the example above, the highlighted part is a false stared, then the speaker corrected themselves.

-Self disruptions:

Self-disruptions and disfluencies in children's speech**.**

specify each type of disruptions according to the following categories:

Cut offs: cutting off oneself mid-sentence/word, producing a single syllable that has no meaning is also considered a cut off (because the word was not completed).

Example for mid-sentence cut off:

"the dog was walking across the bridge and|, with meat in his mouth".

At the mark "|", after the word "and", the child cut themselves mid sentence.

Example for mid-word cut off:

"Then he [tr], then it stung him so he tried to slap it".

The part [tr] is the word "tried" that was cut off.

Example for a meaningless single syllable:

"Then he stopped to at the [s] his shadow".

The sound [s] is a meaningless syllable and considered a cut off.

Mark each cut-off with the code CO.

Pauses: pauses are marked in the transcripts as three dots or more "…". If three dots (or more) are present, it means the speaker paused for 3 seconds or more.

Example:

"it looks like he was gardening and… he sat by the tree".

Mark each pause with the code P.

Repetitions: repeating a sentence, a phrase, a word or part of it, either immediately, or after a self-cut off. Do not code if a word is repeated many times and is clearly repeated on purpose and not a disfluency.

Example of an immediate repetition:

"He looked down on his own reflection. And saw, saw another dog".

The word "saw" is an immediate repetition.

Example of a repetition after a cut off:

"Then he [tr], then it stung him so he tried to slap it".

The word highlighted in yellow is a repetition of the partial word [tr], it was not an immediate repetition, but after a cut off.

Mark each repetition with the code R.

Fillers: filler words that have no meaning on its own (um, uh, ah, erm, well, like, so)

Code: F

Example:

"Um… once there was a man"

Notes: "Well" is not considered a filler if it means "good". "So" is not considered a filler if it can be replaced by "then" or "therefore". "Oh" is not a filler since it has a function of expressing surprise or awe.

Mark each filler with the code "F".

Element 7- Semantic mistakes: the use of the wrong or less precise vocabulary

Example:

"A bald man was standing under a tree after a long day of work".

In this example the participant said standing instead of sitting.

Mark each semantic mistake with the code SM.

Element 8- Phonological mistakes: using the wrong sound in a word, omitting a sound, swapping sounds in a word, using similar sounding words or that rhyme but have a completely different meaning.

Example:

"With a piece of meat in his moud".
The child said moud instead of mouth.

Mark each phonological mistake with the code PM.

Element 9- Morphosyntactic (grammar) mistakes:

Grammatical mistakes: wrong tense, mixing tenses, wrong pronouns, wrong use of "the", "a", and "an", omitting part of a sentence (verb/object/subject), wrong word order. Mark any grammatically faulty structure, even if pragmatically acceptable or revised.

Example:

"The fly stunged him".

In the example above, the participant produced an error in the past tense conjugation.

Mark each grammatical mistake with the code GM.

Table S3

Reliability Coefficients for the Narrative Variables

Variable	Krippendorff's alpha/ ICC
Off-topic deviations	.95
Achronological presentation of events	.79
Events presented	.96
Basic facts presented	.93
Story units presented	.95
Characters/objects introduced	.97
Elaborations to engage the listener	.97
Global coherence rating[a]	.97
Referencing errors	.99
Cohesion errors	.94
False starts	1.00
Self-interruptions	.82
Pauses	.99
Repetitions	.92
Fillers	.96
Phonological errors	.95
Semantic errors	.90
Morphosyntactic errors	.95

[a] = interrater reliability for ratings of global coherence was calculated using Intraclass correlation coefficient ICC.

Table S4

Correlation Coefficients for Narrative Variables and Estimated IQ for the ADHD and NT Groups, and for the Total Sample

Variable	Condition	ADHD<br>*n* = 50	NT<br>*n* = 45	Combined Groups<br>*n* = 95
Informativeness	VC	.06	.03	.10
	noVC	-.16	-.13	-.03
Extra Details	VC	-.13	.18	.06
	noVC	.05	.24	.14
Coherence	VC	-.03	.15	.10
	noVC	.04	.15	.18
Off-Topic Deviations	VC	-.09	-.13	-.13
	noVC	-.06	-.06	-.08
Self-Interruptions	VC	-.19	-.09	-.20
	noVC	.05	-.12	-.10
Pauses	VC	.04	-.05	-.09
	noVC	**.28***	.06	.07
Repetitions	VC	-.10	-.12	-.16
	noVC	.06	-.16	-.09
Fillers	VC	.09	**.42****	**.28****
	noVC	**.35***	**.30***	**.32****
Referencing Errors	VC	-.10	-.08	-.14
	noVC	.17	.11	.05
Cohesion Errors	VC	-.10	-.29	**-.23***
	noVC	.06	-.16	-.12
Grammar and Semantic Errors	VC	-.08	-.18	**-.24***
	noVC	.20	-.24	-.01
Phonological Errors	VC	.26	.04	.08
	noVC	.23	-.06	.09

Note. Correlation coefficients were calculated using Pearson's *r*. VC = visual cue, noVC = no visual cue
$^{**}p < .01$; $^{*}p < .05$

Table S5

Correlation Coefficients for Narrative Variables and CELF-5 Scores for the ADHD and NT Groups, and for the Total Sample

Variable	Condition	ADHD $n = 50$	NT $n = 45$	Combined Groups $n = 95$
Informativeness	VC	.25	.15	**.29****
	noVC	.15	-.20	.16
Extra Details	VC	.03	.19	12
	noVC	.15	-.04	.09
Coherence	VC	**.29***	.16	**.31****
	noVC	**.35***	.13	**.36****
Off-Topic Deviations	VC	**-.38****	-.08	**-.28****
	noVC	-.24	.08	-.16
Self-Interruptions	VC	-.27	**-.37***	**-.33****
	noVC	-.22	-.29	**-.32****
Pauses	VC	-.23	-.15	**-.30****
	noVC	.06	.06	-.09
Repetitions	VC	-.27	**-.32***	**-.30****
	noVC	-.20	-.29	**-.30****
Fillers	VC	.10	**.33***	**.24***
	noVC	**.34***	**.35***	**.36****
Referencing Errors	VC	-.17	.22	-.16
	noVC	**-.35***	-.10	**-.29****
Cohesion Errors	VC	-.22	-.27	**-.23****
	noVC	-.22	-.15	**-.31****
Grammar and Semantic Errors	VC	-.20	**-.37***	**-.35****
	noVC	-.22	**-.40****	**-.34****
Phonological Errors	VC	.09	-.05	-.05
	noVC	.08	**-.32***	-.05

Note. Correlation coefficients were calculated using Pearson's *r.* VC = visual cue, noVC = no visual cue

**$p < .01$; *$p < .05$

Table S6

Correlation Coefficients for Narrative Variables and Working Memory Scores for the ADHD and NT Groups, and for the Total Sample

Variable	Condition	ADHD *n* = 50	NT *n* = 45	Combined Groups *n* = 95
Informativeness	VC	.02	.14	.17
	noVC	-.11	-.01	.10
Extra Details	VC	.12	.05	.11
	noVC	.27	.05	.17
Coherence	VC	.12	.12	**.20***
	noVC	.16	.15	**.30****
Off-Topic Deviations	VC	-.13	-.16	-.18
	noVC	-.15	-.08	-.16
Self-Interruptions	VC	**-.28***	-.26	**-.29****
	noVC	**-.32***	-.25	**-.36****
Pauses	VC	-.07	-.28	**-.26***
	noVC	-.01	-.05	-.18
Repetitions	VC	-.25	-.25	**-.26***
	noVC	-.22	-.20	**-.27****
Fillers	VC	-.05	**.30***	**.17**
	noVC	-.05	.24	**.21***
Referencing Errors	VC	-.21	.16	-.10
	noVC	-.16	-.16	-.21*
Cohesion Errors	VC	.13	-.24	**-.14**
	noVC	-.06	**-.38***	**-.30****
Grammar and Semantic Errors	VC	.07	**-.38****	**-.24***
	noVC	-.13	-.22	**-.28****
Phonological Errors	VC	.25	-.12	.01
	noVC	-.01	**-.38***	-.13

Note. Correlation coefficients were calculated using Pearson's *r*. VC = visual cue, noVC = no visual cue
$^{**}p < .01$; $^{*}p < .05$

Fig.S3

Mean Informativeness and Coherence Scores and Standard Errors for ADHD and NT Groups, Under Visual Cue and no Cue Conditions

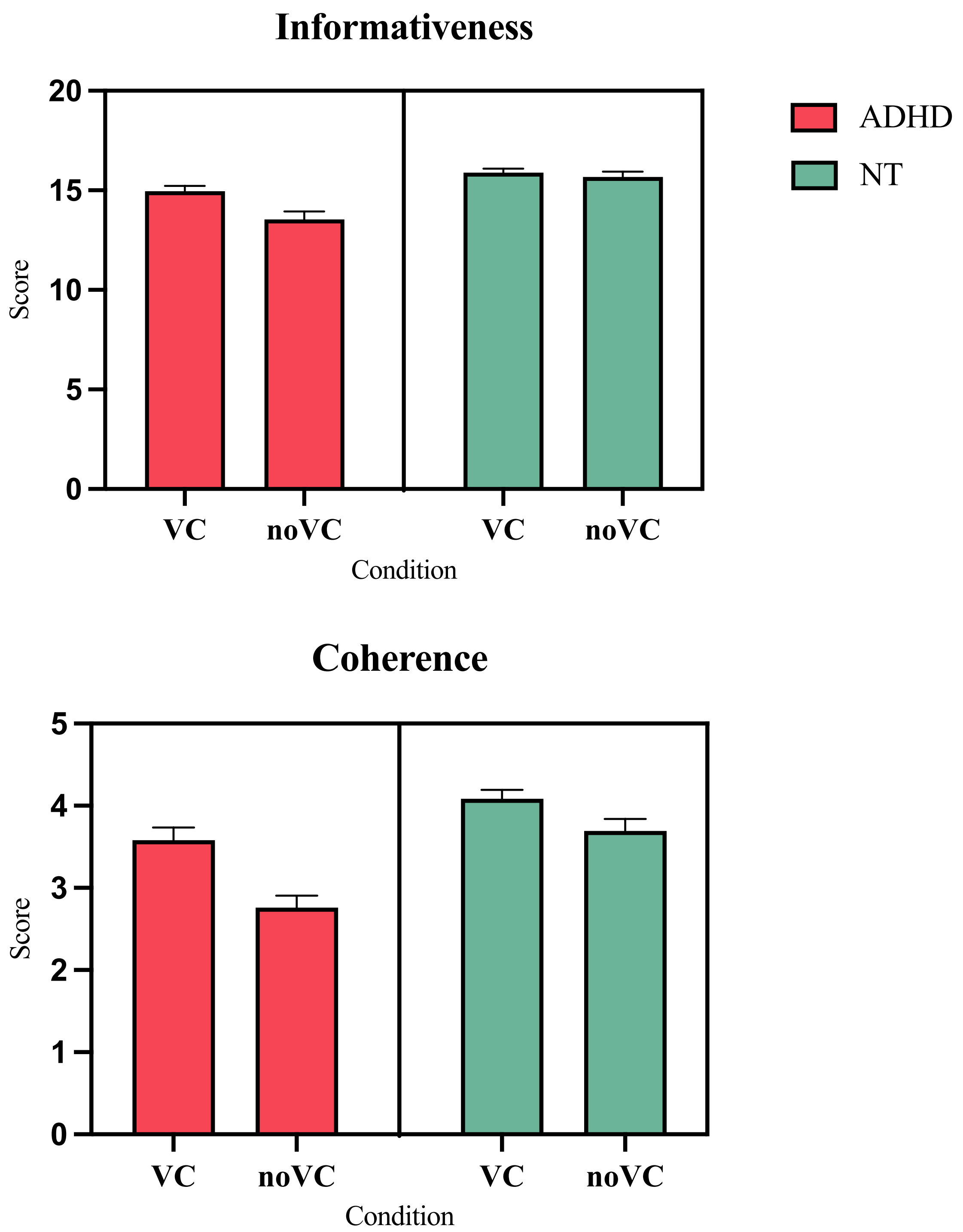

Note. VC = visual cue condition, noVC = no visual cue condition
